# Supplementary figures and images for: Phenotype-based probabilistic analysis of heterogeneous responses to cancer drugs and their combination efficacy
Source: PLoS Comput Biol. 2020 Feb 21;16(2):e1007688. doi: 10.1371/journal.pcbi.1007688 (PMC7055924; doi:10.1371/journal.pcbi.1007688)

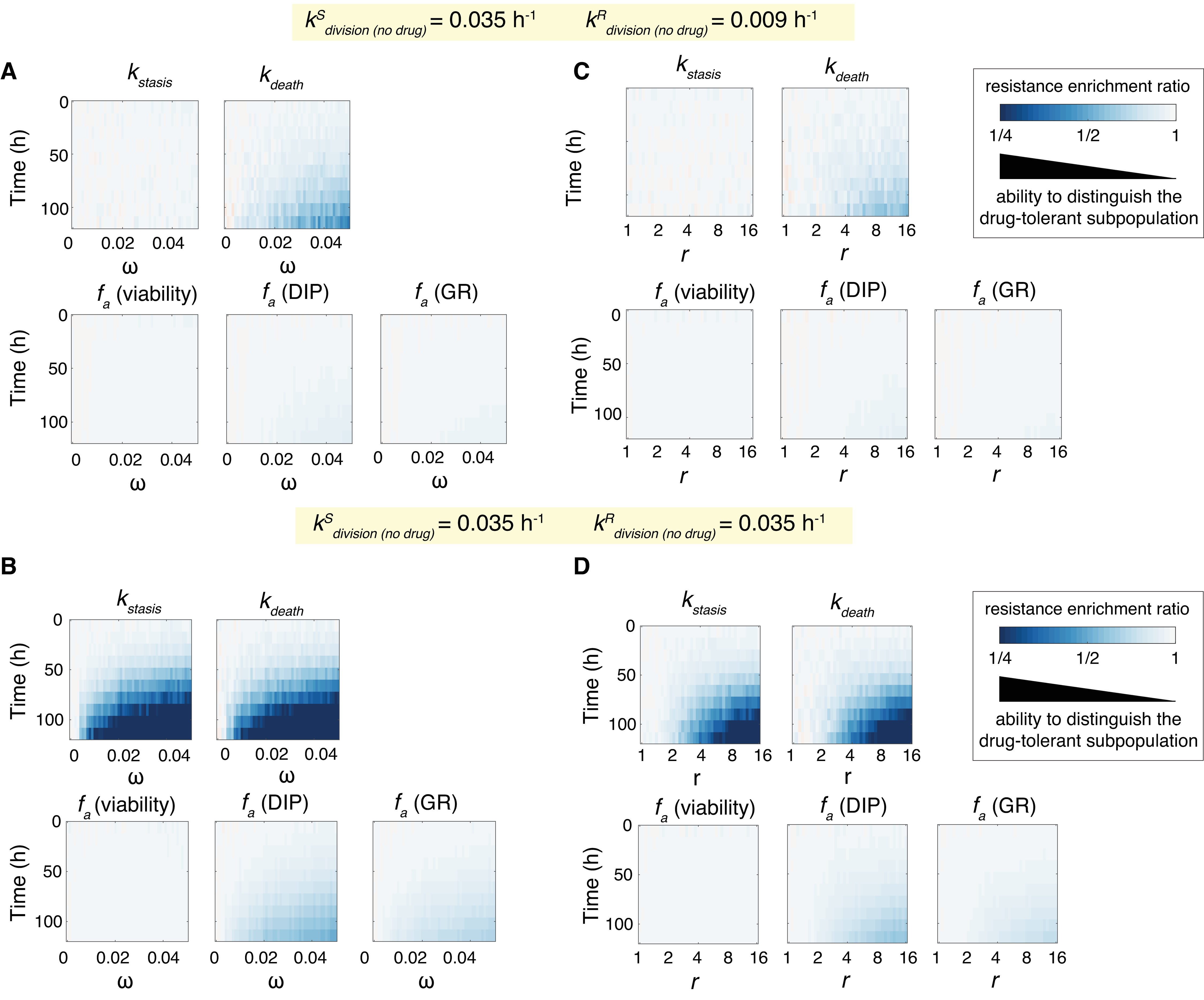

Supplement: S1 Fig — (A,B) Simulation results showing changes in resistant enrichment ratio calculated for each of the fa metrics and for phenotype rate constants (kdeath and kstasis) as a function of ω at a fixed value of r = 16 across different times of treatment. Data are shown for fixed inherent growth rates for the sensitive population, kSdivision (no drug) = 0.035 h-1 and two different rates of inherent growth for the resistant subpopulation: kRdivision (no drug) = 0.009 h-1 (A) and kRdivision (no drug) = 0.035 h-1 (B). (C,D) Simulation results showing changes in resistant enrichment ratio calculated for each of the fa metrics and for phenotype rate constants (kdeath and kstasis) as a function of r at a fixed value of ω = 0.03 across different times of treatment. Data are shown for fixed inherent growth rates for the sensitive population, kSdivision (no drug) = 0.035 h-1 and two different rates of inherent growth rate for the resistant subpopulation: kRdivision (no drug) = 0.009 h-1 (C) and kRdivision (no drug) = 0.035 h-1 (D). All data represent mean values from 50 simulations. (TIF) [file pcbi.1007688.s001.tif]

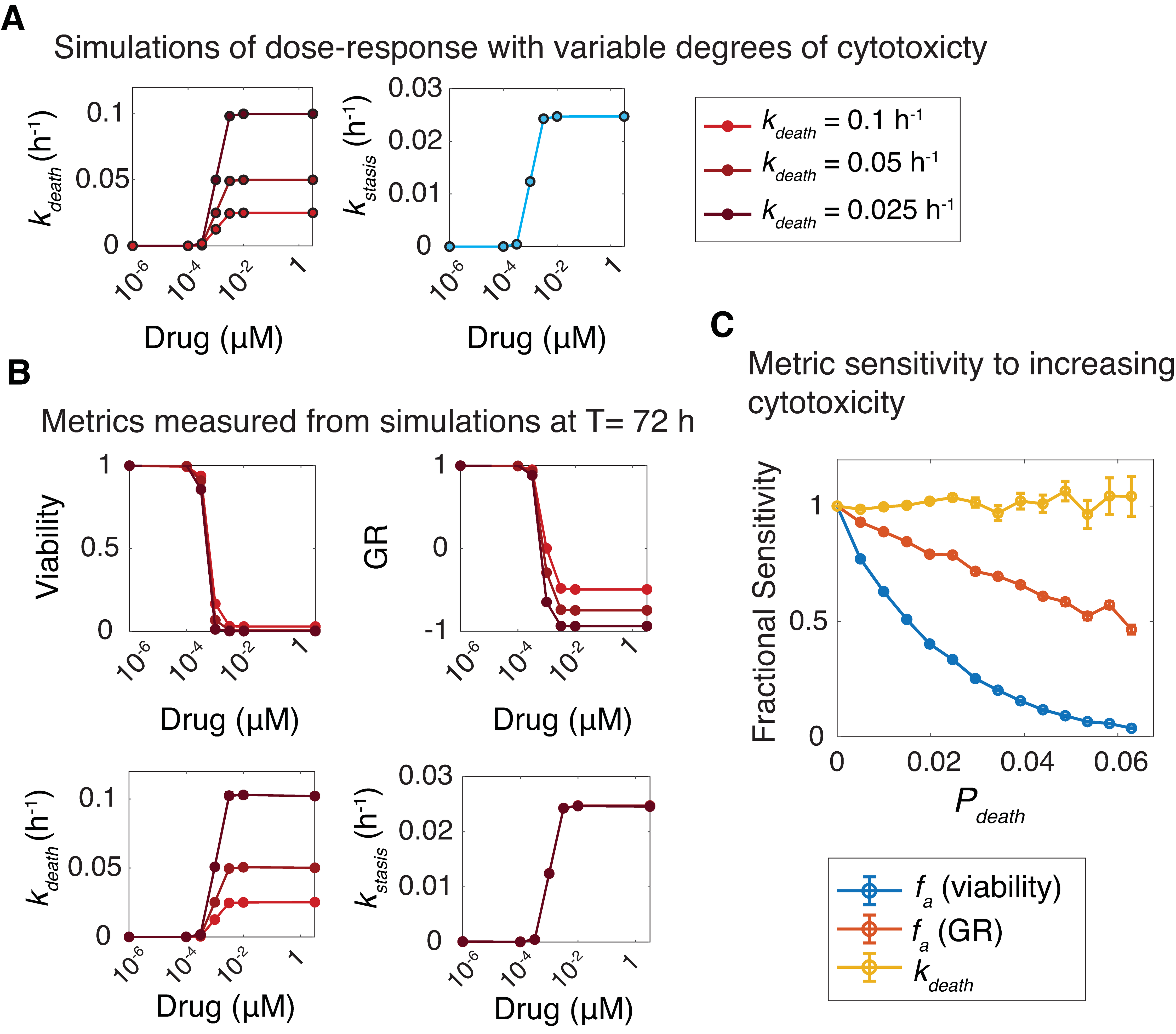

Supplement: S2 Fig — (A) Input dose response profiles used in simulations. The maximum cytotoxic efficacy was varied at three different levels, whereas the cytostatic dose response profiles for all three conditions were held constant. (B) Model output measured from the simulated conditions in (A) at t = 72 h showing variations in viability, GR and the probabilistic phenotype rate constants. (C) Analysis of metric sensitivity with varying drug cytotoxicity parameter Pdeath, quantified per unit of time (h). Sensitivity analysis was performed on simulations with Pstasis = 0 and kdivision (no drug) = 0.035 h-1. Initial cell number was Nlive(t = 0) = 5000. Data shown are mean ± SEM across 50 simulations. Probabilistic phenotype rate constants were estimated from a 24 h time-interval centered at 72 h. (TIF) [file pcbi.1007688.s002.tif]

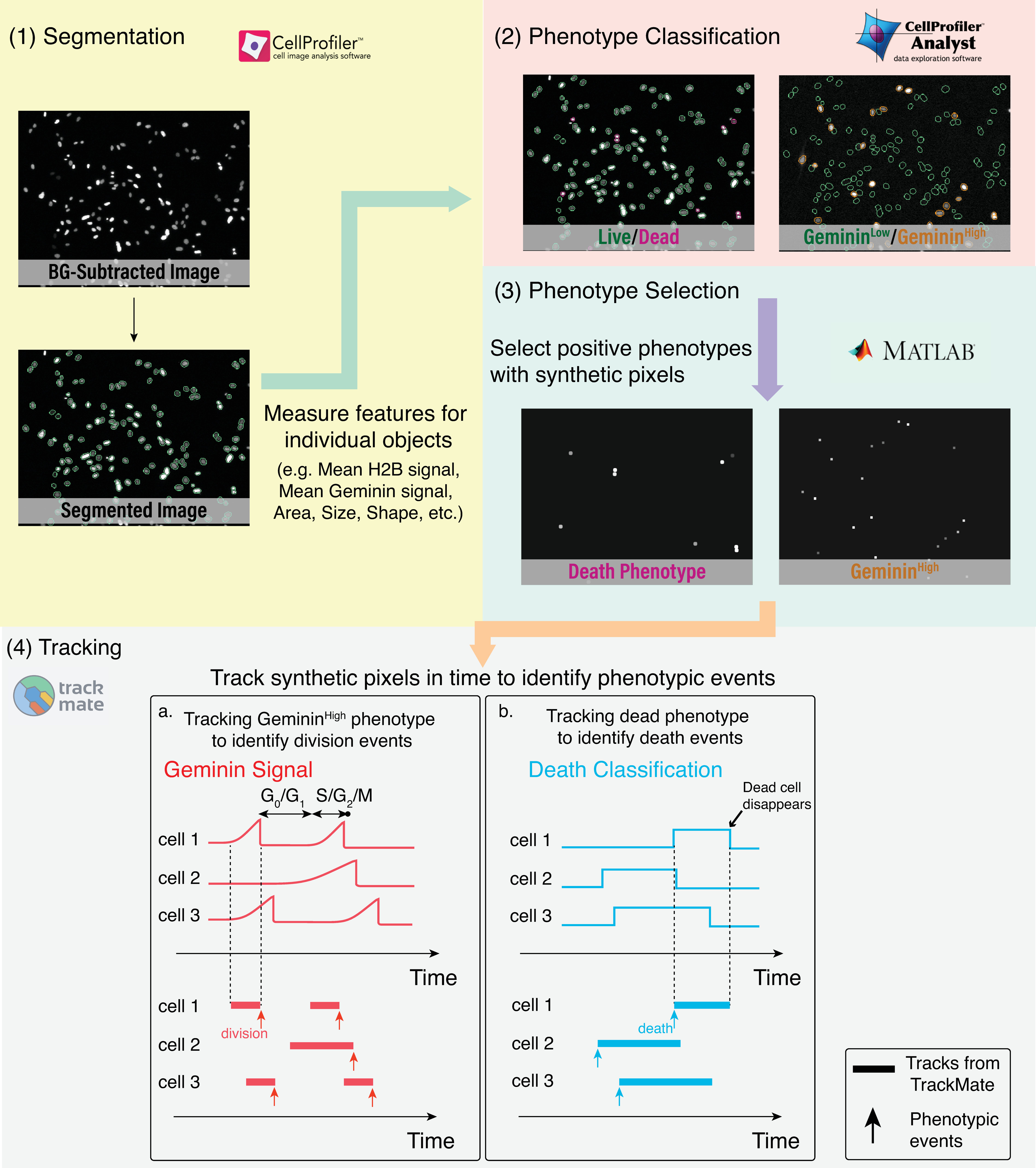

Supplement: S3 Fig — The automated image analysis pipeline involves four steps: (1) Each background (BG) subtracted H2B image was segmented in CellProfiler for nucleus identification. For each nucleus object, a variety of features (e.g. mean signal intensities across multiple channels, area and shape) were measured. (2) To classify the phenotypes of interest (i.e. live or dead cells, Gemininhigh or Gemininlow cells) in each image, classification models were trained in CellProfiler Analyst based on feature measurements of the user-annotated training sets. (3) Based on phenotype classifications of individual cells for each image output from CellProfiler, corresponding synthetic images were generated in MATLAB for each phenotype of interest. Synthetic images contained synthetic pixels at locations of Gemininhigh or dead cells. To facilitate tracking of individual cells, relative intensities of the synthetic pixels for each phenotype were scaled with the mean intensity of the signal associated with that phenotype. For example, intensities of death synthetic pixels were scaled with the mean H2B signal intensities of individual cells, whereas intensities of the Gemininhigh synthetic pixels were scaled with the mean Geminin signal intensities. (4) Synthetic pixels for each phenotype were tracked separately in TrackMate. Since Geminin reporter level drops at the M phase, a division event is marked when the Geminin track ends. The beginning of a death track is also marked as a death event. (TIF) [file pcbi.1007688.s003.tif]

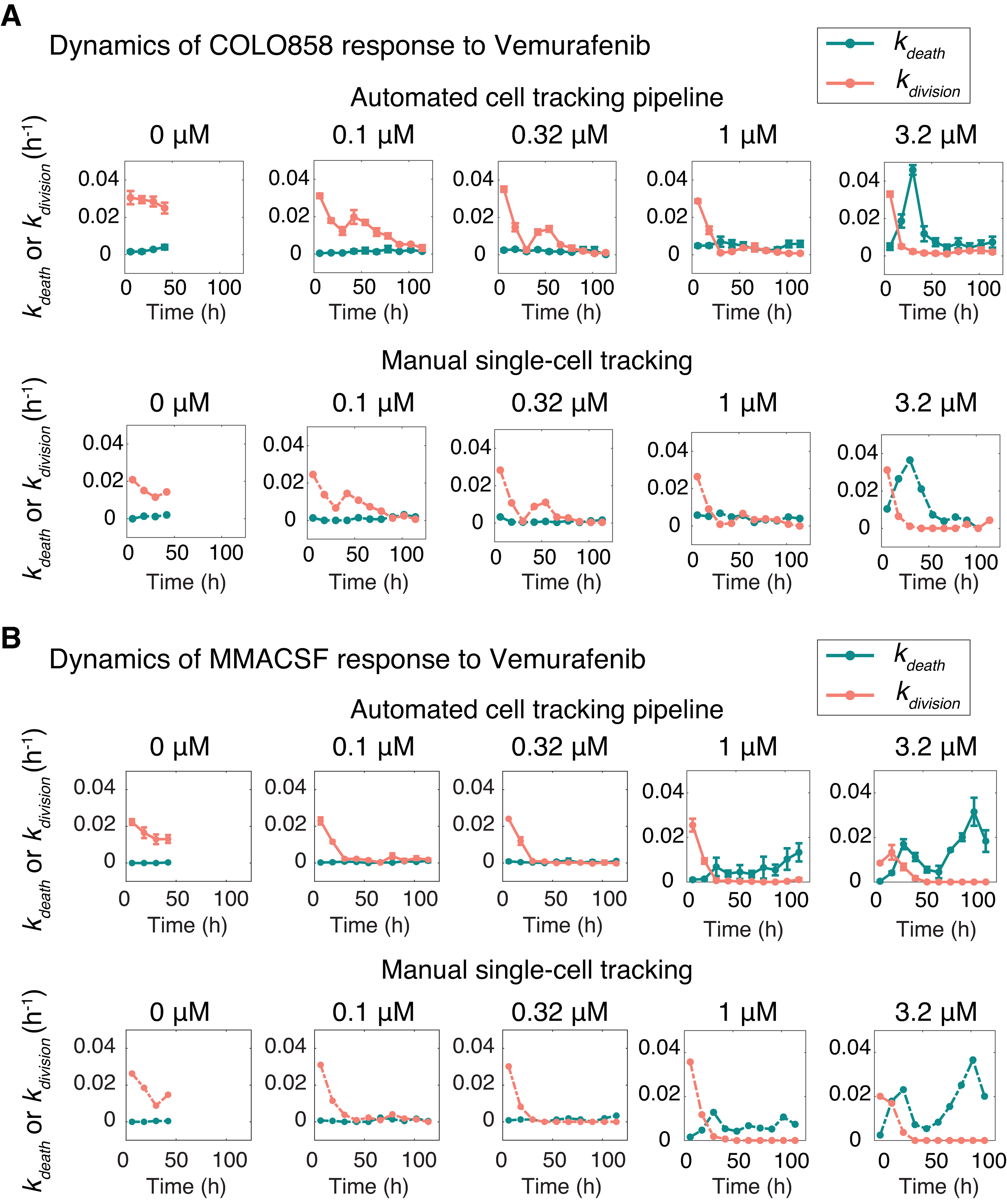

Supplement: S4 Fig — (A-B) Probabilistic rate constants of death (kdeath) and division events (kdivision) measured in (A) COLO858 and (B) MMACSF cells treated with Vemurafenib at the indicated doses, using automated tracking analysis pipeline (top row) versus manual tracking (bottom row) on the same set of time-lapse images. For each condition, the automated tracking estimates at each timepoint are the mean values across four replicated wells. Error bars represent SEM. The rate constants calculated from manual tracking data are based on individually tracked cells pooled from four replicated wells, including about 150–220 cells per condition. (TIF) [file pcbi.1007688.s004.tif]

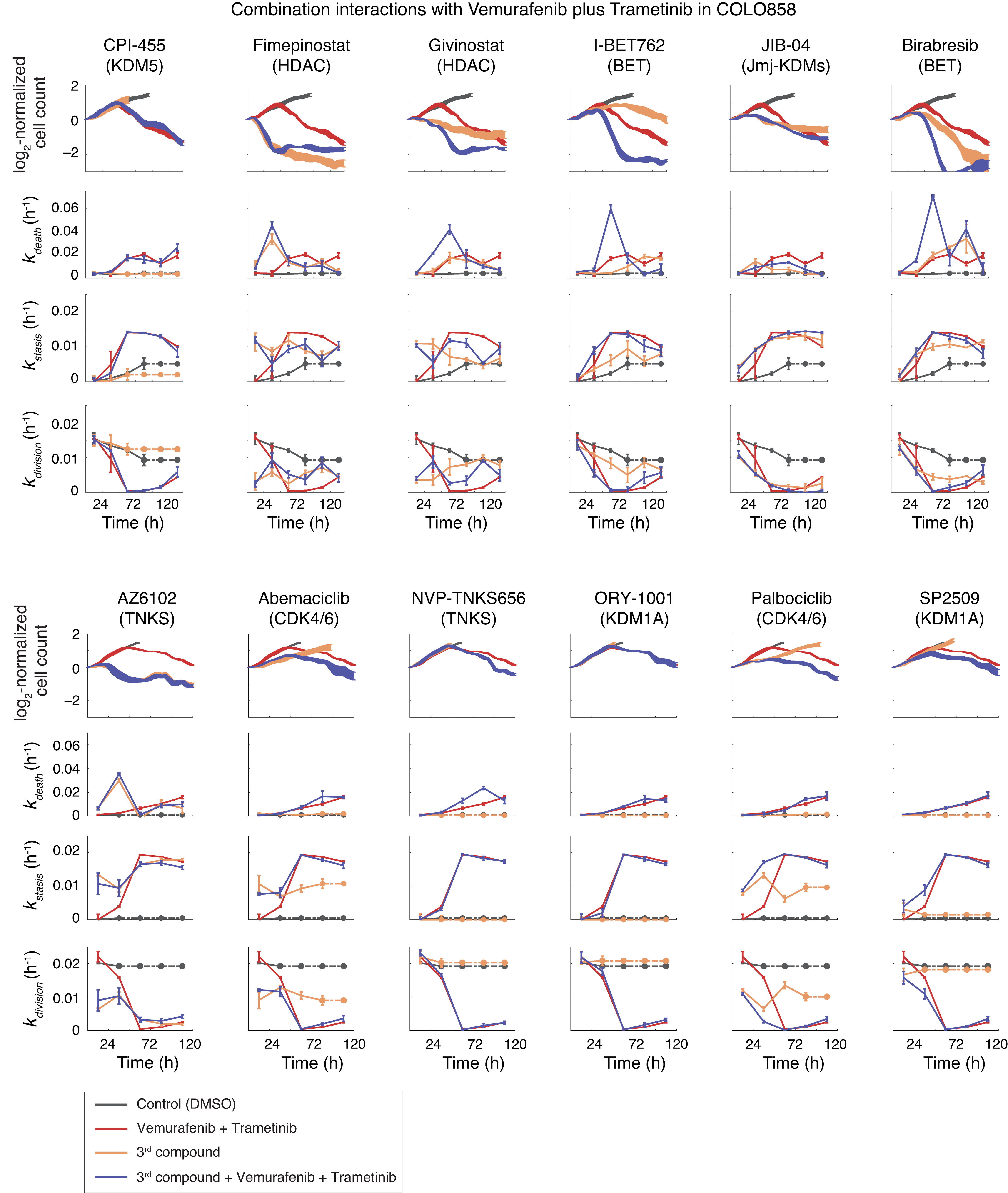

Supplement: S5 Fig — Estimated dynamics of changes in live cell count, kdeath, kstasis and kdivision measured from time-lapse live cell microscopy data for COLO858 cell responses to the combination of Vemurafenib (0.32 μM) and Trametinib (0.032 μM), a 3rd compound (including epigenetic-modifying compounds or cell cycle inhibitors), their triple combination, or vehicle (DMSO) control. Cells were treated initially for 24 h with DMSO control or one of the epigenetic-modifying compounds or cell cycle inhibitors (3rd compound) at the following concentrations: Fimepinostat (0.02 μM), Givinostat (0.2 μM), Birabresib (0.5 μM), I-BET762 (1 μM), SP2509 (1 μM), ORY-1001 (1 μM), JIB-04 (0.2 μM), CPI-455 (5 μM), AZ6102 (1 μM), NVP-TNKS656 (1 μM), Palbociclib (1 μM), and Abemaciclib (1 μM). After 24 h, Vemurafenib at 0.3 μM plus Trametinib at 0.03 μM, or DMSO control were added to each treatment condition. kdivision (no drug) used for the estimation of kstasis is estimated using cell division data averaged for the first 24 h in cells treated with DMSO only. In conditions where confluency was achieved, data-points were replaced with the last available data-point (dotted line). Data-points represent mean ± SEM across 2 or 3 replicates. (TIF) [file pcbi.1007688.s005.tif]

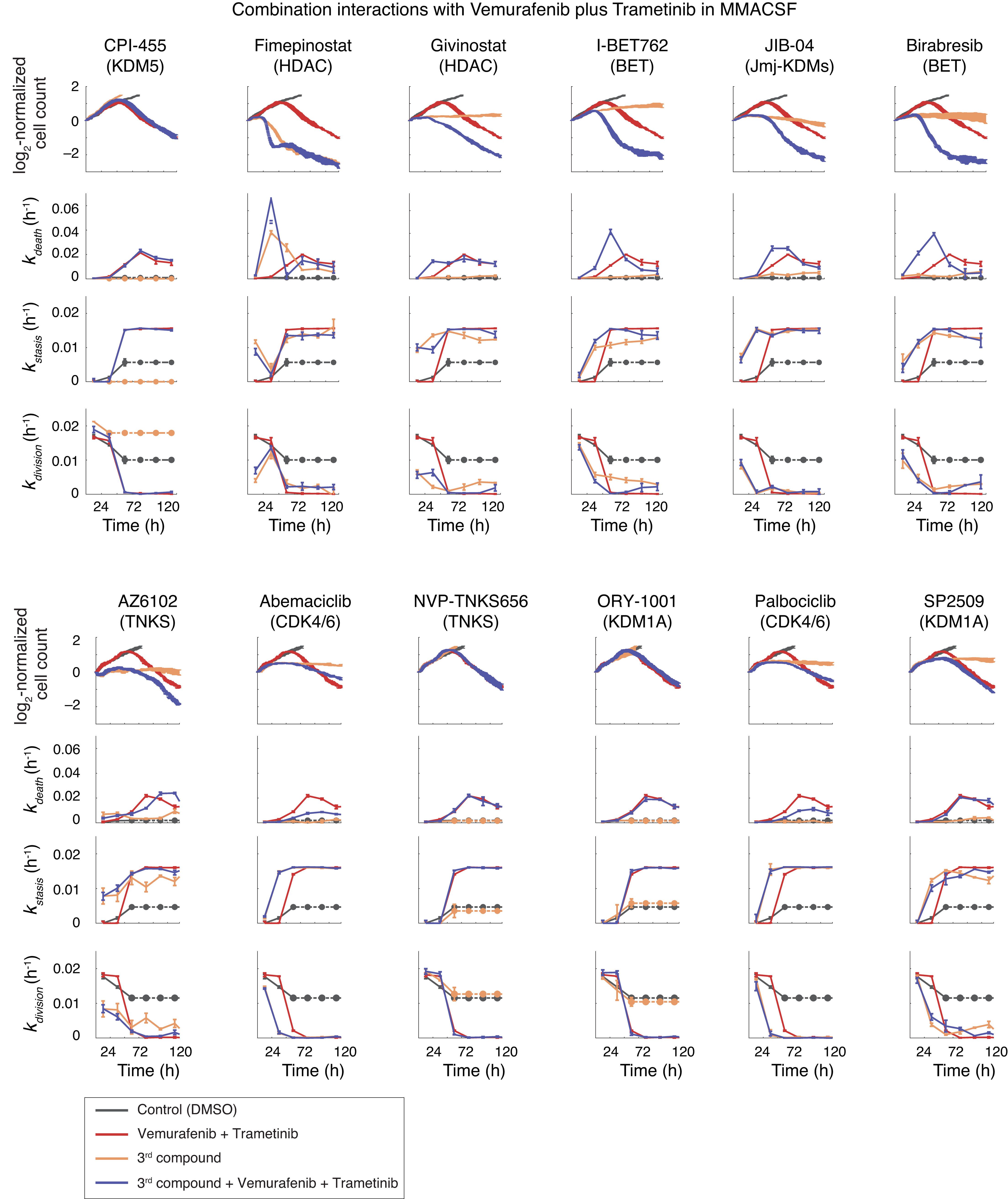

Supplement: S6 Fig — Estimated dynamics of changes in live cell count, kdeath, kstasis and kdivision measured from time-lapse live cell microscopy data for MMACSF cell responses to the combination of Vemurafenib (0.32 μM) and Trametinib (0.032 μM), a 3rd compound (including epigenetic-modifying compounds or cell cycle inhibitors), their triple combination, or vehicle (DMSO) control. Cells were treated initially for 24 h with DMSO control or one of the epigenetic-modifying compounds or cell cycle inhibitors (3rd compound) at the following concentrations: Fimepinostat (0.02 μM), Givinostat (0.2 μM), Birabresib (0.5 μM), I-BET762 (1 μM), SP2509 (1 μM), ORY-1001 (1 μM), JIB-04 (0.2 μM), CPI-455 (5 μM), AZ6102 (1 μM), NVP-TNKS656 (1 μM), Palbociclib (1 μM), and Abemaciclib (1 μM). After 24 h, Vemurafenib at 0.3 μM plus Trametinib at 0.03 μM, or DMSO control were added to each treatment condition. kdivision (no drug) used for the estimation of kstasis is estimated using cell division data averaged for the first 24 h in cells treated with DMSO only. In conditions where confluency was achieved, data-points were replaced with the last available data-point (dotted line). Data-points represent mean ± SEM across 2 or 3 replicates. (TIF) [file pcbi.1007688.s006.tif]
