# Supplementary material for: Phenotype-based probabilistic analysis of heterogeneous responses to cancer drugs and their combination efficacy
Source: PLoS Comput Biol. 2020 Feb 21;16(2):e1007688. doi: 10.1371/journal.pcbi.1007688 (PMC7055924; doi:10.1371/journal.pcbi.1007688)
Supplement: Attachment — Submitted filename: Response_to_Reviewer_Comments_FINAL.docx [file pcbi.1007688.s011.docx]

***Response to Review of PCOMPBIOL-D-19-01684***

***“Phenotype-Based Probabilistic Analysis of Heterogeneous Responses to Cancer Drugs and Their Combination Efficacy”***

We thank the reviewers for their comments and suggestions to improve the manuscript. We have addressed all of the comments in the revised manuscript, where “major” changes are highlighted in yellow. Please find below the detailed, point-by-point response to the concerns raised by the reviewers.

***Reviewer #1:***

**In this study the authors develop a probabilistic description of drug-induced phenotypic events (cytotoxic and cytostatic events). … Comparison of their metrics with the commonly used fa and Bliss independence is well-motivated and should make this paper of interest to many researchers. … The following comments/questions should be addressed/answered to improve the manuscript.**

**The derivation in “Stochastic simulation of cytotoxic and cytostatic drug effects” section assumes that the joint distribution of Pdivision(no drug) Pdivision(with drug) is the same as Pdivision(with drug), that is, a cell would never be more likely to divide with drug than without. Is this true? Are there no treatments that accelerate or force cells through a division? And would this matter? Please clarify.**

The reviewer raises a valid point. It would be theoretically possible that a treatment would accelerate cell division relative to the untreated control. However, the major phenotypic event of interest affected by drug action that we model is the probability of “stasis” (*P_stasis_*) that is defined as the probability with which a drug prevents a cell from dividing given that it would have divided in the absence of drug with a probability of *P_division (no drug)_*. Based on such definition, drugs that do not inhibit cell division and those that accelerate cell division are both characterized by *P_stasis_* = 0. In other words, treatments that enhance tumor cell division are not efficacious in “inhibition of division” and hence do not have any cytostatic effect (*P_stasis_* = 0 and *k_stasis_* = 0). To satisfy this assumption, we consider an upper-bound limit for the probability of cell division that is equal to *P_division (no drug)_*. We have now clarified this point in the manuscript.

**Fixed growth rates for the sensitive and resistant populations were picked to create Figure 2, with the resistant population assumed to grow approximately half as fast as the sensitive population. Can you clarify, does this affect the fa values and how “bad” they appear in terms of resistance enrichment ratio relative to the kstasis and kdeath metrics? It seems like it would matter since the fa metrics depend on time from treatment as well as population growth rate.**

As the reviewer suggested, we have now included simulation results for two additional scenarios using different values of inherent growth rate for drug-resistant subpopulation (*k^R^_division (no drug)_*) relative to the sensitive population. These data are presented in the new Supplementary Figure S1. As expected, the data on resistance enrichment ratio confirms that higher rates of resistant population growth rate are associated with increased sensitivity of all metrics to distinguish the presence of such resistant cells, while the effect is more dramatic for *k_stasis_* and *k_death_* metrics. Overall, the key conclusion that *k_stasis_* and *k_death_* metrics are more sensitive to the presence of drug-tolerant cells than the *f_a_* metrics is consistently true across all values of *k^R^_division (no drug)_*.

**Though it is clear from Figure 2 that the resistance enrichment ratio is smaller for the fa metrics, it isn’t clear to me that that means they aren’t distinguishable. A lower enrichment ratio doesn’t necessary mean that you can’t tell the different between samples, correct? That depends on the (experimental) variability? Perhaps I am missing something here.**

We agree with the reviewer that our choice of words can be improved. In Figure 2, we defined the enrichment ratio to quantitatively compare the theoretical sensitivity of a given metric to heterogenous responses that arise from the presence of drug-tolerant subpopulations. A metric that has greater sensitivity in detecting such heterogeneity (i.e. smaller enrichment ratio) can better capture differences between cases with and without the drug-tolerant subpopulation. As the reviewer pointed out, a key factor here is experimental variability. However, *f_a_* metrics (but not probabilistic rate constants) are also under the influence of the normalization factor (which depends on the behavior of untreated cells) and represent the averaged effect of drug with respect to induction of cell death and inhibition of cell division. We have now revised the manuscript by using the term “sensitivity” instead of “distinguishability”. We have also performed additional sensitivity analysis to directly show this point (see the new Supplementary Figure S2).

**Does Figure 1D necessarily make the point that fa can lead to spurious results? The result we care about is whether or not drug interactions are detected. So the fact that fa metrics don’t necessarily match Pdeath and Pstasis doesn’t necessarily mean that the drug interaction results will be incorrect? We don’t expect fa to be the same as probabilities?**

The goal of the analysis in Figure 1D is to show the quantitative discrepancy of using *f_a_* to represent probabilities of drug effect. This is a key point because *f_a_* metrics are commonly used in “probabilistic evaluation” of drug effect, most importantly in models of drug combination interaction such as Bliss. By rigorously establishing that *f_a_* and probabilities of drug action are not equivalent, we make the point that applying *f_a_* to the Bliss model would lead to unreliable (not necessarily incorrect) conclusions of drug interactions. This point is confirmed later in Figure 4C by systematically simulating different forms of drug interaction.

**The applicability of estimating phenotypic metrics from fluorescently labelled cell lines in time-lapse movies is not clear. The final paragraph of the discussion should be expanded to provide more compelling reasons and applications for this technique.**

We appreciate the reviewer’s suggestion. We have now expanded the last paragraph to include a discussion of important reasons for the application of probabilistic analysis of drug response, current challenges, and how ongoing experimental and computational advances are expected to overcome such challenges.

**Minor comments:
It might be clearer to label Pdivision as Pdivision(with drug) to make clear that this is the probability of cell division in the presence of drug. This is also what is done in the derivation in the Materials and Methods; it would be nice to have the variables be the same everywhere for consistency.**

We have now labeled *P_division_* as *P_division (with drug)_*, showing the probability of division in the presence of drug throughout the manuscript. We did the same with *k_division_* and *k_division (with drug)_*, denoting the rate of division in the presence of drug.

**You say “could be described” on p7 referring to the derivation of Pstasis. Is this because there are other reasonable ways to derive the equation, or because you are making a set of assumptions that are reasonable but might be missing something? Please clarify “could”, and the assumptions that you are making for this derivation either in the main text or in the derivation in the Materials and Methods. This also occurs in the derivation of the Bliss combination index (Eqn 12 and Eqn 13).**

We acknowledge the reviewer’s point regarding the use of a more precise language. The equations for *P_stasis_* and Bliss combination index are derived mathematically or presented based on definition. No additional assumptions beyond what has already been presented in the manuscript have been used to derive any of these equations. We have now revised the manuscript by replacing “could” with more appropriate words.

**The DIP and GR acronyms appear in the main text (p7) without being written out explicitly. I realize that they are in the Materials and Methods (p23) but it would be nice if the reader didn’t have to flip to the Methods to identify the acronym. Similarly, it would be nice for the reader if the equations for DIP and GR were repeated in the Materials and Methods (i.e. steady-state growth rate or first equation in ref. 5) so that we don’t have to go hunt them down in Harris, et al and Hafner, et al. It would also make the necessity of Eqn 17, 18 easier to understand.**

We have addressed these points in the revised manuscript.

**Figure 1D is difficult to understand. I am not completely sure what the easiest way to clarify the figure is? Maybe connecting lines of equal Pdeath (for example in the first figure) instead of having separate disconnected dots?**

As suggested by the reviewer, we have now used dash lines to connect points with equal *P_death_* or *P_stasis_* values in the corresponding figure panels.

**The role of synthetic pixels in the image analysis and tracking program is not completely clear. I read it as synthetic pixels are used to mark dead cells or Geminin-High cells at a single timepoint (or timepoints where those classifications are true). But the how is this connected to the appropriate track of that cell before it died or went Geminin-High?**

As the reviewer correctly points out, synthetic pixels are generated and used to mark each of the dead cells and Geminin^high^ cells in each image that is taken at any timepoint. This is accomplished (in a high-throughput manner) following multiple steps of image segmentation and classification of cellular phenotypes using CellProfiler (3.1.8), as described in detail in Materials and Methods. The goal is to replace the original pixels of H2B and Geminin reporters (which exhibit natural variability from one cell to another) by binary synthetic pixels (representing individual phenotypes of interest) that are then tracked across series of timepoints using the Fiji plugin TrackMate software, without additional need to perform image segmentation to distinguish dead cells or Geminin^high^ cells. In other words, we combine the power of CellProfiler analysis for accurate and high-throughput image segmentation with the ability of TrackMate to track individual cell phenotypes in a multi-day time-lapse experiment. We have revised the relevant Methods section to better explain the role of synthetic pixels.

**In Equation 7, kdivision is ambiguous. Is this kdivision(with drug)?**

Yes. To avoid any confusion, we have now labeled *P_division_* and *k_division_* as *P_division (with drug)_ and k_division (with drug)_* throughout the manuscript.

**Is there a reason the growth timecourse for the control population is so much shorter (72 vs 120 hrs) than the drug treated samples?**

Yes. The untreated COLO858 and MMACSF cells typically reach confluency within the first 60-72 h, after which cell growth and phenotypic parameters are affected. However, drug-treated cells may be analyzed for a longer period of time, depending on drug effectiveness. This point has been clarified in Figure 3 and Figure 5 legends.

**Line on p9 “In particular, as the efficiency of drug-induced cell killing increases, the sensitivity of fa metrics to detect drug tolerance in the surviving fraction of cells decreases”—did you justify this remark?**

Yes. To directly show this point, we have now included sensitivity analysis results for these metrics. The data is presented in the Supplementary Figure S2.

**A very minor point—the comma after your equations keeps moving to the beginning of the next sentence, which is slightly distracting.**

We have removed commas after the equations. We will follow the editorial guidelines for presenting equations in the text.

***Reviewer #2:***

**This manuscript describes a novel mathematical framework for analyzing drug potency in a probabilistic fashion. …I found the approach outlined in this well-written paper to be clever and an advance over the Bliss score currently in use for calculating drug interactions. …The modeling approach works amazingly well. While the methods detail the validation of a non-stationary Poisson process as the underlying assumption for their framework, I still wonder why this is applicable here. Our knowledge of the underlying mechanisms of apoptosis would suggest that the degree of cytotoxicity would initiate release of pro-apoptotic factors that would influence cell death in a manner that isn’t just time-dependent. A more detailed discussion of this would be helpful for the reader to reconcile the appropriateness of the model assumption, especially for different initial seeding densities of cells.**

We appreciate that the reviewer finds the probabilistic approach to heterogenous cancer drug response analysis valuable. The probabilistic modeling approach used in this manuscript involves a commonly used formulation of stochastic chemical kinetics, describing the time evolution of a reacting system while taking into consideration the fact that individual reaction events (typically between individual molecules) are random point *Poisson* processes [1]. While using a similar formulation, we consider a single-cell event (death or division) as an individual event, rather than explicitly modeling any of the drug-induced molecular events that underlie such cellular processes. Previous work (based on live-cell measurement and modeling) has shown that apoptosis, for example, is controlled by a snap action switch at a single-cell level [2]. This switch, however, is associated with a delay that is variable from one cell to another within a population, preventing cells from dying en masse following exposure to a death stimulus. Such variability makes it possible for an all-or-none response at a single-cell level to be graded at the population level such that the concentration of stimulus (or drug) controls the probability with which a cell responds during a specific period of time. This is in general agreement with the definition of *Poisson* process to model the population behavior, where the rate of a cellular event is directly linked to the *Poisson* rate parameter. Such parameter is expected to be controlled by the concentration of a key set of molecular regulators that are not explicitly modeled. However, changes in cellular state (due to drug adaptation, for example) may influence the concentration of these regulators, causing time-dependent changes in the *Poisson* rate constant, making non-stationary *Poisson* process a potentially suitable framework to model such dynamic and adaptive responses. As the reviewer suggested, we have clarified these points and included the explanation regarding the non-stationary *Poisson* process assumption in the Methods section of the manuscript.

**Minor revision, Fig 5A: Hierarchical clustering should be represented with a dendrogram on heat map.**

We have now added the dendrograms corresponding to the hierarchical clustering shown in Figure 5A.

**Have all data underlying the figures and results presented in the manuscript been provided?**
**Reviewer #1: No, Model code and files as well as data extracted from imaging timecourses was not available with my review. These should be made available if the article is published.**

We have now provided the GitHub address where model codes are accessible. Time-lapse microscopy data are provided as part of the Supplementary Information (Tables S1-S4) to the revised manuscript.

**References**

1.	Lecca P. Stochastic chemical kinetics : A review of the modelling and simulation approaches. Biophys Rev. 2013;5: 323–345. doi:10.1007/s12551-013-0122-2

2.	Albeck JG, Burke JM, Spencer SL, Lauffenburger DA, Sorger PK. Modeling a snap-action, variable-delay switch controlling extrinsic cell death. PLoS Biol. 2008;6: 2831–2852. doi:10.1371/journal.pbio.0060299
